# Unique CRF01_AE Gag CTL Epitopes Associated with Lower HIV-Viral Load and Delayed Disease Progression in a Cohort of HIV-Infected Thais

**DOI:** 10.1371/journal.pone.0022680

**Published:** 2011-08-03

**Authors:** Masahiko Mori, Busarawan Sriwanthana, Nuanjun Wichukchinda, Chetsada Boonthimat, Naho Tsuchiya, Toshiyuki Miura, Panita Pathipvanich, Koya Ariyoshi, Pathom Sawanpanyalert

**Affiliations:** 1 Department of Clinical Medicine, Institute of Tropical Medicine (NEKKEN), Nagasaki University, Nagasaki, Japan; 2 Japan Foundation for AIDS Prevention (JFAP), Tokyo, Japan; 3 Thai National Institute of Health, Department of Medical Sciences, Ministry of Public Health, Nonthaburi, Thailand; 4 Global COE Program, Nagasaki University, Nagasaki, Japan; 5 Advanced Clinical Research Center, Institute of Medical Science, University of Tokyo, Tokyo, Japan; 6 Day Care Center, Lampang Hospital, Lampang, Thailand; Karolinska Institutet, Sweden

## Abstract

Cytotoxic T Lymphocytes (CTLs) play a central role in controlling HIV-replication. Although numerous CTL epitopes have been described, most are in subtype B or C infection. Little is known about CTL responses in CRF01_AE infection. Gag CTL responses were investigated in a cohort of 137 treatment-naïve HIV-1 infected Thai patients with high CD4+ T cell counts, using gIFN Enzyme-Linked Immunospot (ELISpot) assays with 15-mer overlapping peptides (OLPs) derived from locally dominant CRF01_AE Gag sequences. 44 OLPs were recognized in 112 (81.8%) individuals. Both the breadth and magnitude of the CTL response, particularly against the p24 region, positively correlated with CD4+ T cell count and inversely correlated with HIV viral load. The breadth of OLP response was also associated with slower progression to antiretroviral therapy initiation. Statistical analysis and single peptide ELISpot assay identified at least 17 significant associations between reactive OLP and HLA in 12 OLP regions; 6 OLP-HLA associations (35.3%) were not compatible with previously reported CTL epitopes, suggesting that these contained new CTL Gag epitopes. A substantial proportion of CTL epitopes in CRF01_AE infection differ from subtype B or C. However, the pattern of protective CTL responses is similar; Gag CTL responses, particularly against p24, control viral replication and slow clinical progression.

## Introduction

Cytotoxic T-Lymphocytes (CTLs) are an important component of the adaptive immune system which mediate control of HIV replication during acute infection and consequent viral set point [1]. Numerous CTL epitopes have been reported across the HIV proteome. However, the influence of CTL on clinical outcome varies, as their recognition of viral antigen is restricted by highly polymorphic class I Human Leukocyte Antigen (HLA) molecules [2], [3]. Furthermore, the tremendous degree of viral diversity increases this complexity; to date, 13 prototype HIV clades and 43 circulating recombinant forms (CRF) have been described [4]. Some epitopes have been reported in a single clade; others have been reported in multiple clades (cross-clade) [5], [6]. No reported epitope to date universally covers all HIV subtypes, or overcomes the global variation in HLA allele distribution (CTL Epitopes. Los Alamos National Lab. http://www.hiv.lanl.gov/).

Gag CTL responses, but not other CTL responses, have consistently been reported to have a significant association with viral control and clinical outcome [7]. However these findings were derived mainly from African or Caucasian populations infected with subtype C or B HIV, respectively; data from Asian populations infected with subtypes circulating in south-east Asia, such as CRF01_AE, have not yet been reported. To determine whether a similar association exists in south-east Asian subtypes, CTL epitope information is essential. However, as of April 2011, only 26 of 420 known Gag epitopes have been reported in CRF01_AE infection. Recently, the first successful phase III HIV vaccine trial was reported from Thailand [8], although its efficacy was marginal. For the development of a more effective vaccine, we believe it is crucial to accurately understand the influence of sequence variation amongst HIV subtypes, and HLA diversity amongst ethnic groups. To provide more information about CTL epitopes in CRF01_AE infection, we investigated cellular immune responses to Gag overlapping peptides in an HIV-1 CRF01_AE-infected Thai population and evaluated their impact on clinical outcome.

## Methods

### Subjects

This study was approved by the Thai Ministry of Public Health Ethics Committee and was conducted according to set guidelines for research. Written informed consent was obtained after explaining the purpose and expected consequences of the study. Patients were eligible for inclusion if they were chronically HIV-infected and antiretroviral-naïve, with a CD4+ T cell count >200 cells/ul. A total of 137 HIV-1 CRF01_AE infected individuals were recruited at a government referral hospital in Thailand from October 2003 to May 2009. Study subjects were requested to visit the clinic every 3 months and CTL responses were evaluated every 6 months. The study endpoint was initiation of antiretroviral therapy, when their CD4+ T cell count declined below 200 cells/ul.

### Synthetic HIV-1 Gag overlapping peptides

Fifteen-mer overlapping peptides (OLPs) of locally dominant CRF01_AE Gag sequences were designed based on 125 *gag* clonal sequences derived from 45 CRF01_AE infected individuals attending the clinic. All deduced amino-acid sequence data were aligned and the most frequent 15-mer amino-acid sequence was used as the dominant sequence.

Peptides were synthesized by Sigma Genosys (Hokkaido, Japan) with a high purity of >90% as determined by high-pressure liquid chromatography. In total, 98 peptides were synthesized and 20 pools were made by mixing 10 peptides per pool in a 10×10 matrix design so that a single responsible peptide could be identified by detecting the common peptide between two reactive pools, as described previously [9]–[11]. When more than one peptide was recognized, we further confirmed the responsible peptide recognition by individually testing candidate peptides, which were suspected by the matrix method.

### ELISpot assay

1×10^5^ fresh PBMC/well were plated onto multiScreen plates (MAHA54510; Millipore) that had been coated overnight at 4°C with 50 µl of anti-gIFN capture Ab 1-D1-K (2 µg/ml; Mabtech, Ohio, USA). Peptides were added directly to wells at a final concentration of 1 µM in 50 µl of R10 and incubated at 37°C in 5% CO_2_ for 24 hrs. PBMC were stimulated with either medium alone for negative control, 10 µg/ml phytohemagglutinin (PHA; Sigma-Aldrich) for positive control or peptide (1 µM final concentration) for 24 hrs at 37°C. Plates were washed extensively with wash buffer (PBS/Tween20 0.001%), followed by incubation with biotinylated anti-human gIFN mAb (0.5 µg/ml; clone 7-B6-1; Mabtech) in PBS/10% FBS for 2 hrs at 37°C. Following six further washes with wash buffer, 2 µg/ml streptavidin HRP (Mabtech) was added to wells with 1 hr incubation at room temperature. Spots were visualized using BCIP/NBT substrate (Chemicon, Australia) and were counted using an Automated Enzyme-Linked Immunospot (ELISpot) Reader System with KS 4.3 software by an independent scientist in a blinded fashion. Each assay was undertaken in triplicate. Spot forming units (SFU) were counted and expressed as SFU per million PBMCs, using the average result from triplicate wells followed by subtraction of the negative control values. A response was defined as positive if it was three times higher than the negative control and greater than 150 SFU/1×10^6^ PBMC. The breadth of response was defined as the total number of peptides recognized by each subject. The magnitude of response for an individual was defined as the sum of all positive peptide responses (in SFU/1×10^6^ PBMC). To avoid overestimation of breadth or magnitude, two adjacent positive overlapping peptides were counted as one response, using the higher of the two responses.

### HLA class I typing

Genomic DNA was extracted from buffy coat using the QIAamp DNA blood Mini Kit (Qiagen, Hilden, Germany) and 4-digit HLA class I typing for A, B and Cw loci was undertaken by bead-based array hybridization (WAKFlow HLA typing kit, Wakunaga Pharmaceutical, Hiroshima, Japan) according to manufacturer's instructions at a commercial laboratory (Kyoto HLA Laboratory, Kyoto, Japan).

### Statistical analysis

Statistical analysis was performed using EXCEL 2007 and SPSS. We first selected viral loads (VL) in the lowest ( = q1) and highest ( = q4) quartiles (n = 34 for each) and compared the number of individuals with positive ELISpot responses to p17, p24 and p15 proteins, using Fisher's exact test to compare groups. We then analyzed the association between breadth and clinical outcome (CD4+ T cell count and VL), using the Kruskal-Wallis test, and between magnitude and clinical outcome (CD4+ T cell count and VL) using Spearman's correlation test. We also performed a longitudinal analysis of the effect of breadth on Highly Active Anti-Retroviral Therapy (HAART) initiation, using the log rank test and Cox regression. For this analysis, the first individual was enrolled on 6 July 2000 and the last individual on 4 September 2007, with a censoring date of 31 May 2009. Analysis of OLP-HLA associations was undertaken using Fisher's exact test with 95% confidential intervals (CI). To have enough statistical power, we analyzed OLP-HLA associations when OLPs were recognized by 3 or more individuals with relevant HLA alleles and at least in one individual, the OLP recognition was confirmed by single peptide ELISpot experiments.

## Results

### Individuals' background, including HLA distribution

Of 137 individuals recruited, 107 were female and 30 were male. Median age was 31 years (range 16–56), CD4+ T cell count 461 cells/ul (range 204–1,191), and VL 4.22 log copies/ml (range 2.60–5.88). No individual had any HIV-related symptoms at the time of enrollment. In total, 87 variations of HLA alleles were found: 23 variations in HLA_A, 46 in HLA_B and 18 in HLA_Cw in four digits (Table S1). Median duration of follow-up was 22 months (range 0–60) and ELISpot experiments were repeated median 4 times (range 1–11) per individual. The peptide recognition pattern was confirmed to be consistent on at least two occasions for all except 24 individuals, in whom ELISpot assays were undertaken only once. During the follow-up period, the peptide recognition pattern did not change in any individual.

### Gag OLP recognition and clinical outcome

**Figure 1:**
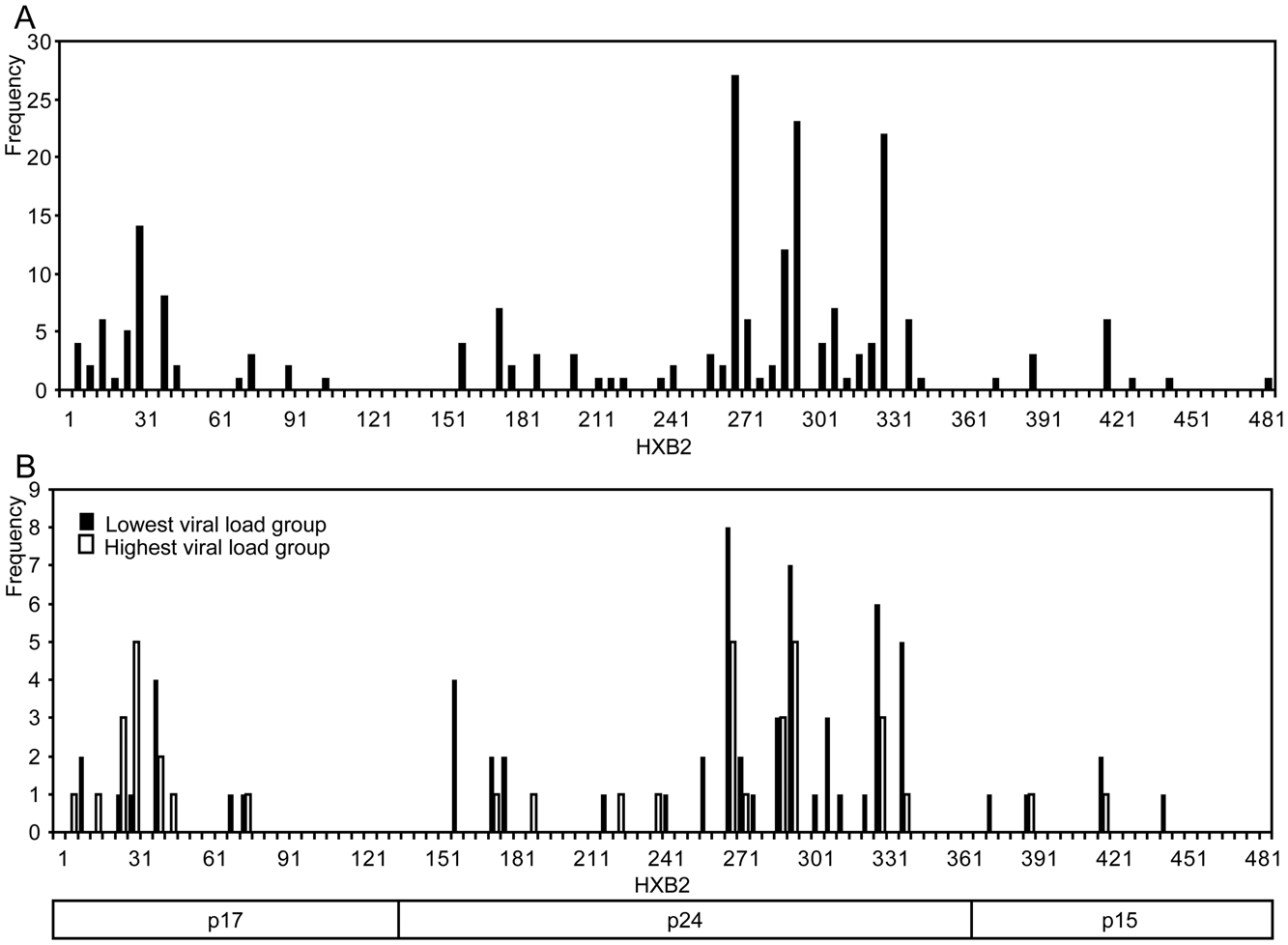
Pattern of CRF01_AE Gag CTL responses. Frequencies of overlapping peptide (OLP) responses in 112 individuals are shown (A); Frequencies of OLP responses in the lowest viral load group (lowest quartile, n = 34) and the highest viral load group (highest quartile, n = 34) were compared (B).

Among 137 individuals, 112 (81.8%) recognized at least one OLP. Of 98 OLPs, 44 (44.9%) were recognized by at least one individual (Figure 1A): 12 peptides in p17, 26 in p24 and 6 in p15. The second half of p24 (HXB2 261–360; OLP 52–69), was the most highly targeted protein region; the first half of p17 (HXB2 5–60; OLP 1–9) was the second most highly targeted region. 14 OLPs were recognized in one individual and the other 30 OLPs were recognized in more than one individual. The most frequently recognized peptides were all located in the second half of p24: OLP 54 (HXB2 271–285), was recognized by 27 individuals; OLP 59 (HXB2 296–310) by 23 individuals; and OLP 66 (HXB2 331–345) by 22 individuals.

To further elucidate the peptide recognition pattern that best contributes to viral control, we next compared ELISpot responses between two extreme VL groups: the lowest quartile ( = q1) (median VL 3.27 log copies/ml (range 2.60–3.71)) and the highest quartile ( = q4) (median 5.09 log copies/ml (range 4.76–5.88)) (Figure 1B). Median CD4+ T cell count was 515 cells/ul (range 243–1,057) in q1 and 429 cells/ul (range 204–856) in q4 (p = 0.022). Interestingly, individuals in q1 more frequently recognized p24 peptides than those in q4 (29/34 vs 18/34, respectively; p = 0.0018, Fisher's exact test), whereas individuals in q4 tended to recognize p17 peptides more frequently (9/34 vs 12/34, respectively; p = 0.6), although this difference was not significant.

### ELISpot breadth, magnitude and clinical outcome

**Figure 2:**
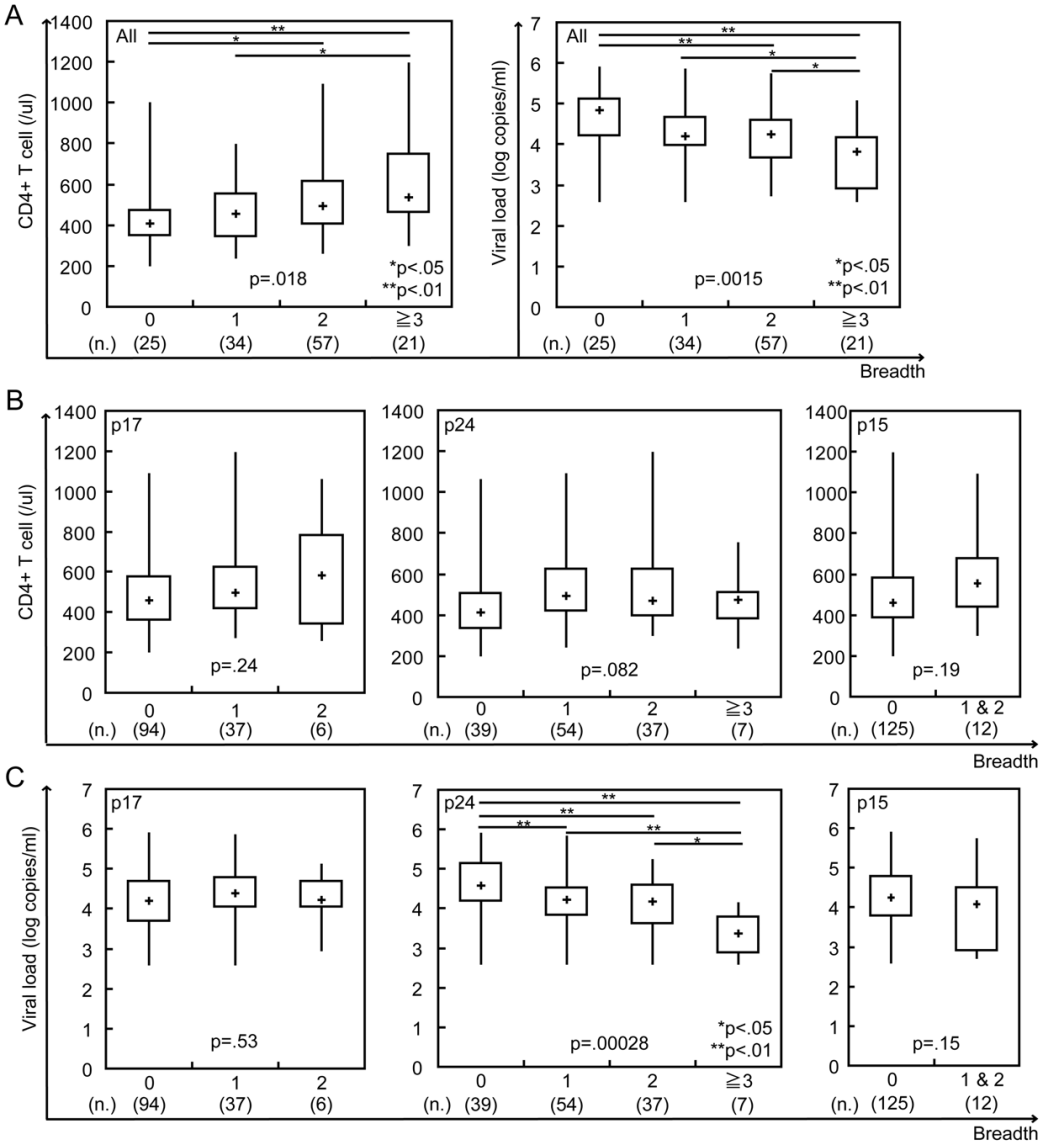
ELISpot breadth is associated with CD4+ T cell count and viral load. The associations between ELISpot breadth (the number of reacting OLP) and CD4+ T cell count or viral load were analyzed using the Kruskal-Wallis test (A). The p17, p24 or p15 site-specific ELISpot breadth was also compared with CD4+ T cell count (B) and viral load (C); * and ** showed a significant difference of p<0.05(*) and p<0.01(**) by Mann-Whitney u-test.

We next investigated the relationship between breadth and clinical outcome. The CD4+ T cell count was significantly higher in individuals with a greater breadth of response, with median CD4+ T cell count of 409 cells/ul (range 204–995), 455 cells/ul (range 243–793), 495 cells/ul (range 264–1,087) and 538 cells/ul (range 303–1,191) in individuals with 0, 1, 2 and ≥3 responses, respectively (p = 0.018 by Kruskal-Wallis test) (Figure 2A left). VL was significantly lower in individuals with a greater breadth of response, with median VL of 4.83 log copies/ml (range 2.60–5.88), 4.21 log copies/ml (range 2.60–5.83), 4.26 log copies/ml (range 2.76–5.71) and 3.82 log copies/ml (range 2.60–5.04) in individuals with 0, 1, 2 and ≥3 responses, respectively (p = 0.0015) (Figure 2A right). In a site-specific analysis, we did not find any significant association with CD4+ T cell count in any sites (Figure 2B). Interestingly, we found a significant association with VL only in p24 (4.57 log copies/ml (range 2.60–5.88), 4.21 log copies/ml (range 2.60–5.80), 4.17 log copies/ml (range 2.60–5.23) and 3.37 log copies/ml (range 2.60–4.14) in individuals with 0, 1, 2 and ≥3 responses, respectively; p = 0.00028) but not in other sites (Figure 2C).

**Figure 3:**
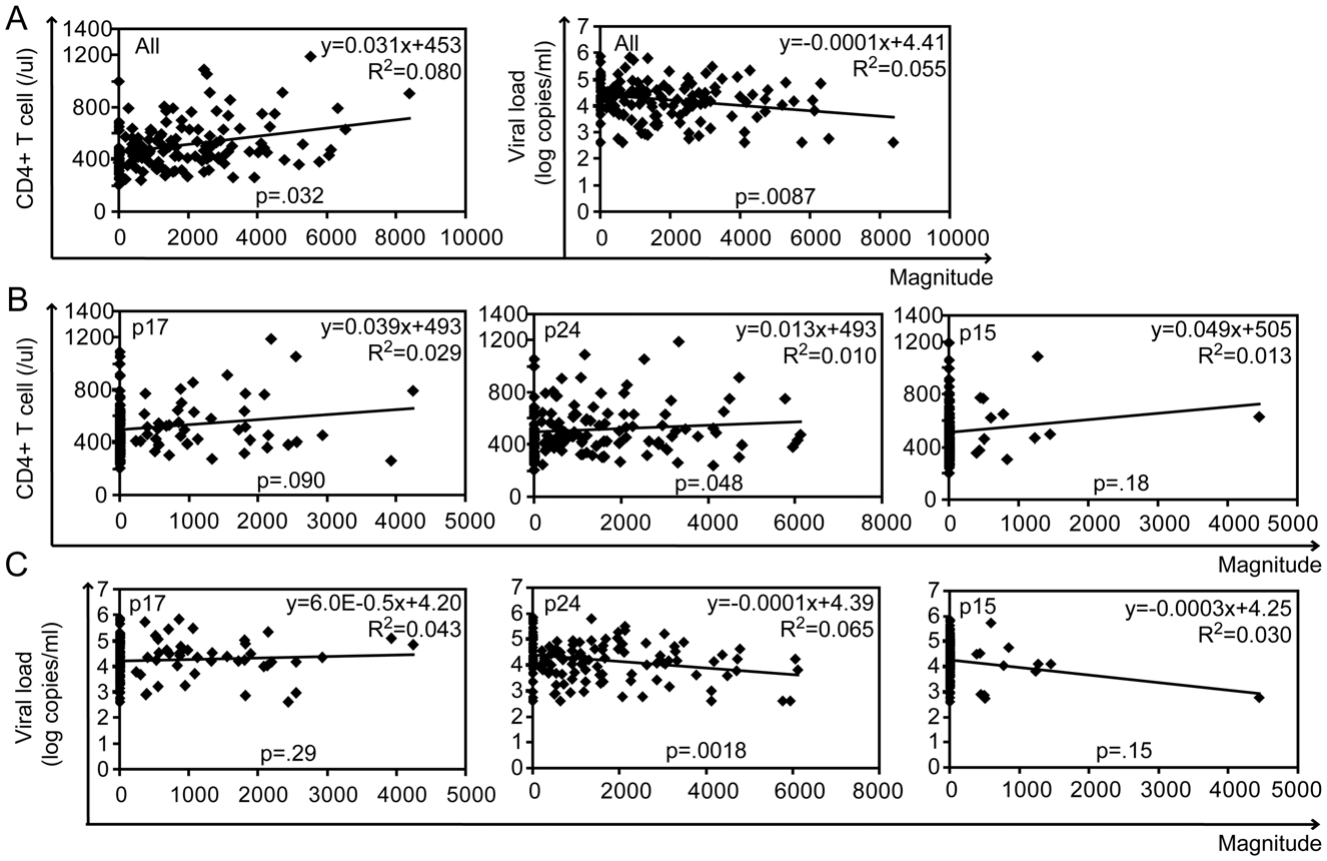
ELISpot magnitude is associated with CD4+ T cell count and viral load. The associations between ELISpot magnitude (total SFU per 1.0 M PBMC) and CD4+ T cell count or viral load were analyzed by Spearman's correlation (A). The p17, p24 or p15 site-specific response was also compared with CD4+ T cell count (B) and viral load (C).

We also found that magnitude of ELISpot response was positively correlated with CD4+ T cell count (p = 0.0032 by Spearman's correlation test y = 0.031x+453 R^2^ = 0.080) and inversely correlated with VL (p = 0.0084 y = −0.0001x+4.41 R^2^ = 0.055) (Figure 3A). In a detailed site-specific analysis, magnitude in p24 had a significant correlation with clinical outcome both in CD4+ T cell count (p = 0.048 y = 0.013x+493 R^2^ = 0.010) (Figure 3B) and VL (p = 0.0018 y = −0.0001x+4.39 R^2^ = 0.065) (Figure 3C), but not in other sites.

**Figure 4:**
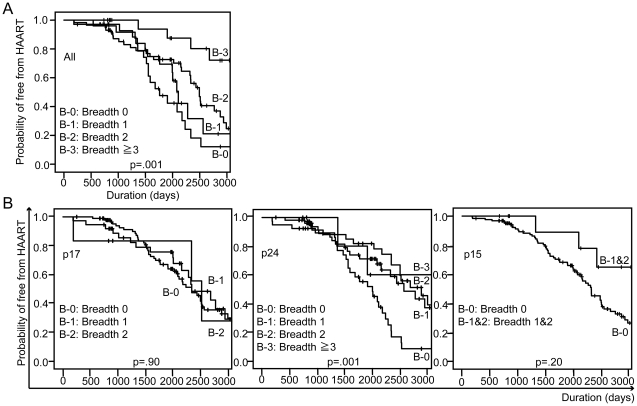
ELISpot breadth is related to delayed initiation of antiretroviral therapy. The impact of ELISpot breadth on antiretroviral therapy initiation was evaluated by Kaplan-Meier analysis, using the log rank test (A). The effect of p17, p24 or p15 site-specific ELISpot breadth was also analyzed (B).

We next investigated the effect of breadth on clinical progression using the initiation of antiretroviral therapy as the end-point. During the follow-up period, 66/137 (48.2%) individuals started antiretroviral therapy. Intriguingly, we found that individuals with a wider breadth of CTL response were less likely to start antiretroviral therapy than those with a narrower breadth of response (Figure 4A, p = 0.001 by log rank test): 18/25 (72.0%), 13/34 (38.2%), 30/57 (52.6%) and 5/21(23.8%) individuals with 0, 1, 2 and ≥3 responses, respectively, initiating antiretroviral therapy. These data imply that strong CTL responses delay clinical progression by slowing the decline in CD4+ T cell count. In a detailed site-specific analysis, individuals with a p24 response, but not other responses, were significantly less likely to start antiretroviral therapy than individuals without a p24 response (p = 0.001). However, the breadth of p24 response did not seem to correlate with clinical progression (Figure 4B).

Multivariate analysis of the relationship between CTL response and initiation of antiretroviral therapy, using Cox proportional hazards model, showed that the association between breadth of CTL response and initiation of HAART was independent of the baseline CD4+ T cell count (>350 cells/ul or not) and VL (<4.0 log copies/ml, 4.0–4.9 log copies/ml and ≥5.0 log copies/ml): adjusted Hazard Ratio (aHR) for individuals making ≥3 OLP responses was 0.23 (p = 0.005 with 95% CI of 0.08–0.64).

### Detection of reactive OLP-HLA association

Associations between OLP responses and HLA were statistically analyzed. In total, 14 peptides (4 in p17, 9 in p24 and 1 in p15) with 31 OLP-HLA associations were identified (Table S2). 13 associations were found both with HLA-B and Cw alleles each and 5 were found with HLA-A alleles. Two adjacent OLPs shared the same responsible HLA allele: HLA_A*0207, B*4601 and Cw*0102 in OLP 54–55, and B*4601 in OLP 58–59, suggesting that CTL epitopes reside in the overlapping region of these peptides. Some of the OLP-HLA associations may not reflect genuine CTL epitopes. 10 OLP responses were associated with two or more responsible HLA alleles. Of these, 9 OLP responses were associated with a pair of HLA alleles in linkage disequilibrium (LD), which were identified using the Los Alamos database (HLA Linkage Disequilibrium. Los Alamos National Lab. http://www.hiv.lanl.gov/). Among the 10 OLP responses, 7 included reported epitopes in either one of the HLA alleles. OLP 54, 55 and 59 responses were also associated with HLA alleles that have haplotype associations: HLA_A*0207-B*4601-Cw*0102. In total, 11 OLP-HLA associations were compatible with previously reported CTL epitopes: 4 epitopes were already reported as cross-clade epitopes including CRF01_AE or subtype A and the remaining 7 epitopes were reported in other subtypes but neither in subtype A nor CRF01_AE. Consequently, we identified at least 17 OLP-HLA associations in 12 OLP regions; 6 OLP-HLA associations (35.3%) were not compatible with previously reported CTL epitopes, suggesting that these are likely to contain unique CRF01_AE Gag CTL epitopes.

## Discussion

This is the first study to investigate Gag CTL epitopes and their effect on clinical outcome in a systematic way in a CRF01_AE-infected Asian cohort. In this study, which tested optimal OLPs in a well-described cohort, we succeeded in predicting a number of unique CRF01_AE Gag epitope and novel cross-clade epitope candidates. Although one third of CTL epitope candidates in CRF01_AE infection were not compatible with previously reported CTL epitopes in other subtypes, both cross-sectional and longitudinal analysis showed the pattern of protective CTL responses was similar to previous studies; specifically, that a Gag CTL response, particularly against p24, was associated with better control of viral replication and slower clinical progression [7], [11]–[15]. These findings are also compatible with our previous study in which an association with clinical outcome was found only for the number of HLA-associated mutations in p24 but not in other sites [16]. Both studies imply that immune pressure on p24 Gag influences the clinical outcome in CRF01_AE infected Asian individuals. Several papers have discussed the advantages of CTL immune pressure against p24 for viral control , which include selection of escape mutations that lead to viral fitness cost [17], [18], sequence stability compared with other viral particles [4], [19], [20], the abundance of Gag protein in incoming virions [21], and more rapid antigen presentation of Gag epitopes following viral infection [18].

While our findings showed the clear-cut relationship between ELISpot breadth and clinical parameters, the slopes of the trend lines between ELISpot magnitude and clinical parameters were rather shallow. Furthermore ELISpot magnitude did not correlate with onset of HAART initiation. These findings are consistent with a recently published study that breadth of the CTL response rather than magnitude associated best with clinical outcome [22].

In this study, we could not detect any OLP-HLA associations in HLA_B*57, which is well-known as one of the most protective alleles for viral control [2], [3], [23]. Three individuals expressed B*5701; however, none had any response to OLP 47, which contains the TW10 (TSTLQEQIGW) epitope [24]. We have previously found in our cohort that all B*57 patients had the T242N escape mutation [16]. This suggests that the virus circulating in B*57 individuals lacks the wild-type TW10 sequence *in vivo* and no longer stimulates TW10 CTL cells [25].

In this study, OLP-HLA associations were predicted by statistical analysis. Thus these associations are not necessarily a reflection of new CTL epitopes with responsible HLA alleles. We excluded LD associations, including haplotypes and adjacent OLP responses with the same HLA allele association, in which CTL epitopes presumably reside in the overlapping region of these peptides. The most immunodominant OLP, number 54 (NKIVRMYSPVSILDI), was associated with three HLA alleles: A*0207, B*4601 and Cw*0102. “RMYSPVSIL” was previously identified as an A*0207-restricted CTL epitope [26]. All three responsible HLA alleles were found to be in LD. However, the association with B*4601 and Cw*0102 was much stronger than for A*0207 (odds ratio 29.4 in B*4601 and 104 in Cw*0102 vs 5.5 in A*0207) and further analysis including by ^51^Cr release assay is warranted.

From this study, we have substantially increased information about CTL epitopes in CRF01_AE infection, reporting at least 6 unique CRF01_AE CTL epitope and 7 novel cross-clade epitope candidates. CRF01_AE is a recombinant HIV-1 with Gag derived from subtype A [4], from which CTL epitope information is limited, compared to subtypes B or C. We anticipate that if a more detailed epitope mapping study were to be conducted in subtype A-infected populations, there would be a large number of epitopes cross-recognized between CRF01_AE and subtype A.

Although details of OLP-HLA associations are substantially different between subtypes, interestingly we found a similarity in the immunodominant regions between subtypes. Our data showed that the second half of p24 was the most immunodominant regions, followed by the first half of p17 regions. This finding is consistent with previous reports [13], [15], [27]. We were concerned that the compatibility between OLP sequences and circulating Gag sequences may vary depending on the conservativeness and influence on the pattern of Gag CTL responses. However, the proportion of gag clones that were completely matched to the amino-acid sequence of OLPs was not associated with the frequency of OLP responses (data not shown).

Cross-clade CTL responses are said to be influenced by the viral sequence variability between subtypes, especially the sequence at anchor positions of the HLA binding motif [4], [28]–[31]. Among the 7 newly identified cross-clade epitope candidates, 6 shared the same sequences with reported epitopes at both the B and F pockets. We also compared sequence compatibility at the anchor positions of the best-defined 12 epitopes, not identified in our study. 11 out of 12 also had compatible sequences at anchor positions, implying that sequence compatibility at anchor positions per se does not predict cross-clade reactivity. Other factors should be considered, such as sequences at flanking regions affecting peptide cleavage by the proteasome [32], [33] and epitope-HLA complex recognition by T cell receptors (TCRs) [34], [35].

This study has a number of limitations. First, we focused on Gag CTL immune responses and did not investigate whole viral proteins. However, since this type of analysis requires a large number of cells, and the volume of blood that we were able to take was rather limited, we decided to focus on Gag responses, as Gag is known to be the most important viral target. Instead of testing a large number of OLPs individually, we undertook experiments in triplicate, using a matrix system, to improve reliability. However, it would have been ideal if we had obtained enough volume of blood to confirm all responses using the individual peptides. Second, we detected OLP-HLA associations by a statistical method and not by the standard HLA-restriction analysis. This approach is easily influenced by sample size and the impact of LD. Thus our study does not provide direct evidence. Third, we have not yet confirmed these OLP responses with CTL using the ^51^Cr release assay. However, ELISpot assays are now widely accepted as a technique for mapping CTL epitopes [36]. Fourth, these data are based on single cytokine release of gIFN; we did not evaluate multi-functionality of CTL with other cytokines such as IL2 or TNFα [37].

However, our data indicate the existence of a substantial number of unique CTL epitopes in CRF01_AE infection; it is therefore worth conducting a systematic analysis of CTL epitopes when vaccine trials are undertaken in different populations infected with different subtypes.

## Supporting Information

Table S1 **HLA allele frequencies in the study population.**
(XLS)

Table S2 **Gag overlapping peptide responses and their HLA allele associations.**
(XLS)
